# Drug Repurposing in Non-Small Cell Lung Carcinoma: Old Solutions for New Problems

**DOI:** 10.3390/curroncol30010055

**Published:** 2023-01-05

**Authors:** George Doumat, Darine Daher, Morgan Bou Zerdan, Nasri Nasra, Hisham F. Bahmad, Monica Recine, Robert Poppiti

**Affiliations:** 1 Faculty of Medicine, American University of Beirut, Beirut 1107-2020, Lebanon; 2 Faculty of Medicine, University of Aleppo, Aleppo 15310, Syria; 3 The Arkadi M. Rywlin M.D. Department of Pathology and Laboratory Medicine, Mount Sinai Medical Center, Miami Beach, FL 33140, USA; 4 Department of Translational Medicine, Herbert Wertheim College of Medicine, Florida International University, Miami, FL 33199, USA

**Keywords:** drug repurposing, non-small cell lung cancer, therapy resistance, review

## Abstract

Lung cancer is the second most common cancer and the leading cause of cancer-related deaths in 2022. The majority (80%) of lung cancer cases belong to the non-small cell lung carcinoma (NSCLC) subtype. Despite the increased screening efforts, the median five-year survival of metastatic NSCLC remains low at approximately 3%. Common treatment approaches for NSCLC include surgery, multimodal chemotherapy, and concurrent radio and chemotherapy. NSCLC exhibits high rates of resistance to treatment, driven by its heterogeneity and the plasticity of cancer stem cells (CSCs). Drug repurposing offers a faster and cheaper way to develop new antineoplastic purposes for existing drugs, to help overcome therapy resistance. The decrease in time and funds needed stems from the availability of the pharmacokinetic and pharmacodynamic profiles of the Food and Drug Administration (FDA)-approved drugs to be repurposed. This review provides a synopsis of the drug-repurposing approaches and mechanisms of action of potential candidate drugs used in treating NSCLC, including but not limited to antihypertensives, anti-hyperlipidemics, anti-inflammatory drugs, anti-diabetics, and anti-microbials.

## 1. Introduction

Lung cancer is the leading cause of cancer-related deaths in 2022, with 350 deaths per day [1]. It has the second-highest incidence rate among all cancers, with 236,740 projected new cases in 2022, second only to breast cancer in females and prostate cancer in males [1]. Cigarette smoking is still a major culprit behind lung cancer, as it directly caused 81% of lung cancer death in 2022 [2]. This relationship is reflected in global incidence and mortality trends; regions with lower human development index have higher incidence and mortality from lung cancer [3]. Lung cancer incidence declined during the last decade by 3% in men and 1% in women [1]. This decline narrowed the historic gender gap in lung cancer incidence, with the men to women ratio once reaching three-fold in the 1970s, caused by the disproportionate smoking rates among men and women [1]. Despite having the highest mortality, lung cancer survival is more promising during all stages. The percentage of people living at least three years after diagnosis increased from 19% in 2001 to 31% in 2017, with a median survival increase from 8 to 13 months [1]. The survival gains can be attributed to advanced diagnostic procedures and treatments such as enhanced pathological staging and video-assisted thoracoscopic surgery [4,5].

Lung cancer is broadly classified into small cell lung carcinoma (SCLC) and non-small cell lung carcinoma (NSCLC), with NSCLC accounting for 80% of the cases. Within NSCLC, adenocarcinoma is the most common subtype, accounting for 60% of the cases. Other NSCLCs include squamous cell, large cell, adenosquamous, pleomorphic, spindle cell, and giant cell carcinomas [6].

When assessing the cell of origin of NSCLC, a group of distal adult lung epithelial stem cells called the bronchioalveolar stem cells (BASCs) were identified at the bronchioalveolar duct junction [7]. Even though it was later shown that BASCs do not act similar to tissue stem cells, these cells are pluripotent as they proliferate when a *KRAS* mutant is induced [8]. The expansion of the BASC population in *KRAS*-mutant mice was correlated with tumor progression and an increase in cell size and number [8].

Multiple molecular pathways affecting oncogenes and tumor suppressor genes have been implicated in the pathobiology of the NSCLC [9]. One of the key cancer-related genes affecting NSCLC is the epidermal growth factor receptor (*EGFR*) gene. Mutations in *EGFR* genes are typically diagnosed in adenocarcinoma patients; they are ubiquitous among those with never-smoking status, female gender, and East Asian ethnicity [10]. Another gene commonly identified in adenocarcinoma is the anaplastic lymphoma kinase [11] gene [11], specifically the *EML4-ALK* fusion in young, never-smoker patients [12]. Another common gene detected in adenocarcinoma among smokers is the Kirsten rat sarcoma viral oncogene homolog gene (*KRAS*). The *KRAS* and *EGFR* pathways in adenocarcinoma are mutually exclusive, which suggests different molecular pathways being implicated in the development of adenocarcinoma between smokers and non-smokers [13]. The *MET* gene, which occurs in 7% of NSCLC, encodes for a receptor tyrosine kinase that activates multiple signaling pathways involved in lung cancer pathogenesis and metastasis [14]. The fibroblast growth factor receptor type 1 (*FGFR1*) gene encodes for a cell surface tyrosine kinase receptor of the FGFR tyrosine kinase family. *FGFR1* has been reported to be amplified in 20% of squamous cell carcinomas and 1–3% of adenocarcinomas [11,15]. Finally, mutations in the discoidin domain receptor 2 (*DDR2*) gene, a receptor of tyrosine kinase, were found in 4% of squamous cell carcinomas [16].

The stage of NSCLC at the time of diagnosis is critical in determining survival; supported by the fact that the 5-year relative survival rate for localized NSCLC is 64%, as opposed to 8% for metastatic NSCLC [17]. Staging also directs treatment with the standard of care for stage I cancers being surgery with either lobectomy or pneumonectomy as the procedure of choice [18]. The treatment of stage II cancers includes surgical resection along with cisplatin-based adjuvant systematic therapy [18,19]. The standard of care therapy for unresectable stage III NSCLC since 2017 has been concurrent chemoradiation followed by durvalumab [20,21]. This combination therapy offers a 24-month overall survival rate of 66.3% and a median progression free survival of 17.2 months [21]. Patients with stage III NSCLC are managed with multimodal treatment regimens consisting of concurrent or sequential chemotherapy and radiotherapy [18,19]. The five-year survival in stage IV disease is only 1–3%, so therapy is aimed at alleviating symptoms with single- or double-based chemotherapy [18]. More recent drugs treating NSCLC include targeted therapy, specifically tyrosine kinase inhibitors erlotinib, gefitinib, and afatinib targeting EGFR [22]. Another newly utilized therapy avenue is immunotherapy, which includes IgG4 monoclonal antibodies against programmed cell death protein 1 (PD-1), such as Nivolumab and Pembrolizumab [18].

Cancer stem cells (CSCs), the underlying subpopulations of tumor cells that drive tumor growth and progression, cause NSCLC to be a heterogenous tumor [23]. Cancer stem cells are a subpopulation of cells residing within the tumor bulk that are believed to be responsible for therapy resistance and recurrence [24,25,26,27,28,29,30,31]. This heterogeneity drives drug resistance in NSCLC, leading to therapeutic failure [23]. The plasticity of CSCs in NSCLC allows them to reverse differentiate into different cell types [32]. CSCs play a major role in driving resistance to multiple chemotherapy agents by releasing multidrug ATP-binding cassette (ABC)-transporters [33]. Some of the cell-intrinsic treatment resistance mechanisms in NSCLC include activating pro-survival and anti-apoptotic pathways and signaling and expressing drug transporters [23]. Moreover, the resistance can be classified as on-target resistance, affecting oncogenes such as *ROS1* and *RET* rearrangements and *BRAF* mutations. Resistance can also be off target, affecting signaling pathways via epigenetic modifications [32]. 

Even though the treatment of NSCLC is advancing, the treatment resistance, plasticity of the tumor, and heterogeneity cause treatment to be challenging. So, in addition to exploring novel agents for treating NSCLC, we should turn our attention to repurposing some of the readily available agents to help combat the aggressive disease (Table 1).

## 2. Repurposing Approved Drugs in Cancer

The substantial cost, slow pace, and high attrition rates of new drug discoveries are driving many researchers toward drug repurposing, also known as drug repositioning [60]. Drug repurposing is the use of existing drugs to treat a new medical condition, which was not the intended indication of the drug [61]. The new indication is built upon the established safety, pharmacokinetics, and manufacturing data of existing drugs, which includes approved, discontinued, shelved, and investigational therapeutics [27,28,30,31,61]. 

**Figure 1:**
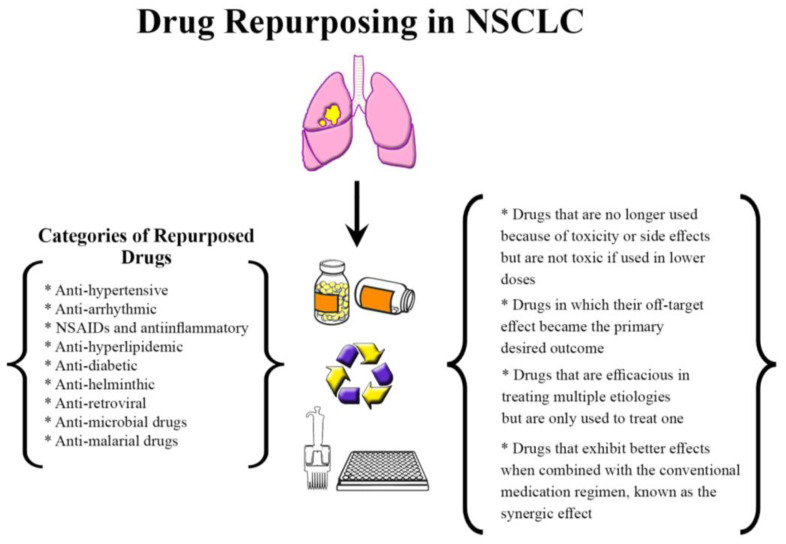
Drug repurposing in NSCLC. Food and Drug Administration (FDA)-approved drugs identified and repurposed to treat patients with NSCLC. Abbreviations: NSAIDs: non-steroidal anti-inflammatory drugs.

Repurposed drugs fall into various classes; first, drugs that are no longer used because of toxicity or side effects but are not toxic if used in lower doses or a different population [62]. An excellent example from this class is thalidomide, which was teratogenic to pregnant women when used for morning sickness but was later found to be safe and efficacious in treating refractory multiple myeloma. Another class of repurposed drugs is the ones in which their off-target effect became the primary desired outcome, such as sildenafil, which was developed as an anti-anginal medication but is now used to treat erectile dysfunction. An additional class includes medications that are efficacious in treating multiple etiologies but are only used to treat one. A final class involves medications that exhibit better effects when combined with the conventional medication regimen, known as the synergic effect [62] (Figure 1).

### 2.1. Computational-Based Approach to Drug Repurposing

Since there are multiple classes of repurposed drugs, there are also multiple approaches to repurposing these drugs. The computational method, also known as in silico drug repurposing, involves the collection and analysis of diverse types of data from different sources, mainly from databases, including chemical structure, gene expression, proteomics, or electronic health records (EHRs) [63]. This approach combines data to locate and explore potential medications for repurposing and involves different techniques.

The first technique of the computational approach is network-based drug repurposing, which utilizes the advances in genotyping technology by using genome-wide association studies (GWAS) to find genetic variants that affect common diseases [64]. GWAS identifies new targets shared by multiple disease phenotypes; however, these targets might not be druggable. Using network-based algorithms allows researchers to find druggable genes upstream or downstream of the target gene [65,66]. Examples of drugs repurposed using this technique include vismodegib, an inhibitor of the Hedgehog signaling pathway, to treat Gorlin syndrome [67], and iloperidone, an antipsychotic for the treatment of schizophrenia, to treat hypertension [68]. Another example includes the random walk propagation algorithm, which expands a set of disease-associated genes to genes sharing neighbors in a gene–gene or protein–protein network. This algorithm was used to identify novel indications for diseases such as gabapentin for anxiety disorder, cisplatin for breast cancer, donepezil for Parkinson’s disease, and methotrexate for Crohn’s [69]. 

Another technique in the computational approach is profile-based drug repurposing, which compares different drug profiles with another drug, disease, or clinical phenotype [70]. An example would be comparing the differential gene expression in a cell or tissue before and after therapy and contrasting it to the expression profile associated with the disease [71,72]. For example, suppose a drug is shown to reverse the transcription of a certain gene related to the pathophysiology of a disease. In that case, the drug can reverse the disease and should be a candidate for repurposing [71,72]. Another profile-based technique compares the chemical structure of different drugs, which can lead to new drug-target associations [73]. This can be performed via molecular docking, which evaluates multiple ligands against a receptor [60]. Mebendazole, an antiparasitic medication, was found to have, using a computational docking technique, the structural capacity to block vascular endothelial growth factor receptor 2 (VEGFR2) [74].

Moreover, retrospective clinical data can be used as a tool in drug repurposing, especially with the abundance of data extracted from EHRs [75]. Raloxifene for breast cancer, propranolol for osteoporosis [76], aspirin for colorectal cancer [77], and sildenafil for erectile dysfunction [78] were all repurposed based on simple clinical studies rather than complex network analyses. Text-mining tools accelerate the data-based drug repurposing approach by decreasing the time needed to go through the complex scientific literature. Text-mining was used to create Alzheimer-specific drug–protein connectivity maps, which found diltiazem and quinidine as possible therapeutic candidates [79].

### 2.2. Pros and Cons of Drug Repurposing

The main advantages of repurposing drugs in treating cancer revolve around time and cost. It was shown that the average time from filing the investigational drug application to the first new drug application is 8.3 years for novel antineoplastics and from 3 to 4 years for repurposed drugs [78,80]. This improved timeline stems from the fact the pharmacokinetics and pharmacodynamics of the repurposed drug are already established, which sometimes overcomes the need for a phase I clinical trial [60]. Another advantage of repurposing drugs is cost. It costs USD 300 million to bring a repurposed drug to the market, compared to the USD 2–3 billion it requires to develop a novel drug [81].

Nevertheless, repurposing drugs has some disadvantages; for example, redirecting an established drug toward new usage is a high-risk investment as the new direction might not be as successful as planned. Additionally, some repurposed drugs might undergo similar processes in terms of time and cost as novel drugs would, to ensure their safety in the new population [60].

## 3. Repurposing Approved Drugs in NSCLC

Oncology has greatly benefited from the recent movement toward drug repurposing, given that only 5% of antineoplastic drugs that enter a phase I trial will eventually be approved [82]. This is driving research groups to repurpose known drugs and use them in the battle against highly resistant cancers, such as colon and prostate cancers [30,31].

Despite improved screening efforts and declining incidence rates, NSCLC still poses many therapeutic challenges. These challenges can be summarized by high resistance to conventional chemotherapy, which causes single-drug therapy to be useless, necessitating double therapy or a combination of chemotherapy and radiotherapy [12,32]. The need for more therapy options causes NSCLC to be a suitable candidate for drug repurposing.

### 3.1. Anti-Hypertensives and Anti-Arrhythmic Drugs

Beta-blockers are a class of drugs known to be useful in the management of cardiovascular diseases, hyperthyroidism, migraines, and glaucoma. With the increasing popularity of drug repurposing, the anticancer effects of beta adreno-blockers are now being studied more extensively. The exact mechanism for their anti-NSCLC effects is still unknown, but many speculations exist. A study by Sidorova et al., which studied the effects of beta blockers on the viability and cell colony formation of NSCLC, showed that propranolol and betaxolol are the most effective in inhibiting lung cancer cell colony formation at 90% of the EC50 value [83]. Propranolol decreases tumor angiogenesis and can stimulate the immune system [84]. Beta blockers may help treat cancer via desensitization of beta receptors to chronic beta-agonist use. This increases IL-6 expression stimulating cell proliferation in lung cancer and inhibits tumor suppressor liver kinase B1 (LKB1) in *EGFR*-positive lung adenocarcinoma tumors [34]. Chronic adrenergic stimulation impairs the response to chemotherapy. Thus, using beta blockers could help enhance the effectiveness of chemotherapy [35]. Oh et al. found that patients treated with immune checkpoint inhibitors and beta-blockers had increased progression-free survival [36]. However, there has been no correlation between using beta-blockers alone and improved overall survival in lung cancer [85]. Another study by Sidora et al. proved the effect of beta-blockers on NSCLC cell apoptosis with no significant difference between selective and non-selective beta-blockers in vitro. However, further investigation is needed concerning their anti-tumor role in vivo [83]. 

Angiotensin I converting enzyme inhibitors (ACEIs) and angiotensin II receptor blockers (ARBs), known to have life-prolonging effects, are frequently used drugs to treat numerous diseases, including hypertension and heart failure [30]. The renin-angiotensin-aldosterone system has been linked to many of the hallmarks of cancer, which is one rationale behind repurposing ARBs and ACEIs to treat NSLC [86]. The interaction between angiotensin II and angiotensin II receptor 1 stimulates vascular smooth muscle cells to produce VEGF, which aids tumor angiogenesis [87]. Telmisartan is an ARB clinically indicated for hypertension therapy and has also proved to have anti-cancer effects by activating PPARγ, which halts tumor metastases [37]. Based on a study by Godugu et al., in vitro experiments with losartan and telmisartan nanoparticles proved to be both effective against lung cancer and well tolerated by human normal fibroblast cells. Due to their adequate aerosol performance, both drugs could be used as inhalation aerosols for lung cancer treatment. However, the intratumoral distribution of telmisartan was around 2.7 times higher than losartan. Hence, telmisartan exhibits a significantly higher anticancer and antifibrotic role in lung tumor models [38].

### 3.2. NSAIDs, Anti-Inflammatory Drugs and Aspirin

Nonsteroidal anti-inflammatory drugs (NSAIDs) have anti-inflammatory, analgesic, and antipyretic properties by inhibiting cyclooxygenase (COX) enzymes [88]. According to recent data, COX-2 is upregulated in lung adenocarcinomas. COX-2 is associated with enhanced cell proliferation and reduced apoptosis, two factors necessary for invasive tumor growth and metastasis. Therefore, COX-2 inhibition forms an important checkpoint [89]. Several NSCLC cases have been associated with the overexpression of COX-2 proteins in lesions of human lung adenocarcinoma and hint at a worse prognosis [90]. Celecoxib is a specific inhibitor of COX-2 widely used initially as an anti-analgesic for osteoarthritis and rheumatoid arthritis pain [91]. The anti-cancer role of celecoxib is through various intrinsic and extrinsic pathways associated with apoptosis and the downregulation of NF-kB, caspase-9, BAX, and BCL-xL [92].

Furthermore, celecoxib binds to 3-phosphoinositide-dependent protein kinase-1 (PDK-1) to inhibit the PDK1/Akt pathway via a COX-independent mechanism. PDK1/Akt controls nucleus–centrosome coupling and regulates microtubules and MAP binding to microtubules. Therefore, by suppressing the PDK1/Akt pathway, the aerosolized formulation of celecoxib should be effective in treating NSCLC [39]. Based on an intervention performed by Haynes et al., inhaled celecoxib resulted in in vitro cytotoxic and apoptotic responses against human NSCLC, demonstrated by the increase in PPAR-γ and p53 expression [41]. In addition, evidence suggests the possible involvement of a COX-2-independent pathway of celecoxib against NSCLC. Under physiological conditions, cPLA2 and 5-LOX regulate the arachidonic acid pathway. By releasing arachidonic acid from membrane lipids, cPLA2 and 5-LOX may promote lung mouse tumorigenesis. However, in the presence of aerosolized celecoxib, cPLA2 and 5-LOX expression in NSCLC cells decreased substantially [40]. In addition, indomethacin [36] is another NSAID that was proven to aid in NSCLC. A few clinical trials have combined this drug with other cytotoxic drugs. IND was noted to induce apoptosis in the cancer cells by initiating the caspase-3 enzymatic activity through upregulating the Bax, Bak, and PPAR-ɣ pathway [42,43]. Sarvepalli et al. prepared a liposomal formulation of IND to increase its anticancer potential. The significant inhibition of COX-2 and induction of caspase in all the IND-treated groups was observed. Sarvepalli et al. concluded that liposomes are more efficacious compared to plain IND in treating NSCLC [89]. However, additional clinical studies and in vivo experiments are required to attain a complete view of this approach and to validate this therapy.

### 3.3. Anti-Hyperlipidemic Drugs

Statins are the drugs of choice for treating elevated cholesterol levels in the blood [93]. Many pre-clinical and clinical findings have proven the anti-neoplastic effects of statins [31]. Thus, drug repurposing studies have focused on statins as an off-label drug for cancer treatment [94]. By inhibiting β-hydroxy β-methylglutaryl-CoA (HMG-CoA) reductase, an enzyme essential for synthesizing cholesterol [95], statins decrease the production of mevalonate derivatives that are essential for many growth regulatory processes such as proliferation, apoptosis, and differentiation [25]. Atorvastatin is a statin with an antitumor mechanism that inhibits Akt/mTOR and activates the MAPK pathway [84]. Atorvastatin induces apoptosis of tumor cells by depleting the isoprenoid driven growth proteins necessary for the function of cell-growth-stimulating proteins Ras, Rac, and Rho. In addition, by stimulating autophagy and ferroptosis, a type of programmed cell death associated with excess iron overload and accumulating lipid reactive oxygen species, atorvastatin acts as an anti-tumor agent [96]. Hosseinimehr et al. studied the effects of atorvastatin on lung cancer cells. In this study, the apoptosis rate of A-549 lung cancer cells that were both treated with atorvastatin and irradiated was higher than the apoptosis rate of cells that were only irradiated; this difference was statistically significant. This was correlated to atorvastatin’s role in producing ROS, which aids in the apoptosis of NSCLC [44,45]. 

Lovastatin is another statin that exhibits a hypolipidemic effect by competing with HMG-CoA reductase. Lovastatin also induces apoptosis by inhibiting cell proliferation and regulating cancer cell signaling pathways [97]. To study lovastatin efficiency in NSCLC, Walther et al. found that lovastatin increases COX2 expression and subsequently activates PPAR-γ, which induces the cytotoxicity of lung cancer cells [98]. 

Simvastatin is another anti-hyperlipidemic being repurposed in the treatment of NSCLC. A phase II trial showed that treating NSCLC with gefitinib plus simvastatin instead of gefitinib showed a higher response rate and prolonged progression-free survival [99]. On a molecular level, Simvastatin was shown to induce apoptosis in *p53* mutated cells, in addition to inhibiting cell growth and proliferation [48].

Furthermore, Pitavastatin can also be used in the treatment of NSCLC because of its proven potential in in vitro studies [47]. Using a combination of Pitavastatin and erlotinib on an *EGFR*-TKI-resistant human lung adenocarcinoma cell line showed a promising synergic cytotoxic effect. The proposed mechanism involves the induction of alternative regulated cell death pathways by inhibiting mevalonic acid (Mev) and the pan-caspase inhibitor zVAD [47]. Its anti-neoplastic effects were also explained by downregulating the MVA pathway and inhibiting the expression of EGFR, hence suppressing the Ras/Raf/MEK/ERK signal cascade and inducing the apoptosis of lung cancer cells [100]. 

To conclude, even though more research is needed to better explain and affirm the effectiveness of statins in treating NSCLC, much research has already correlated the role of statins in ameliorating the treatment outcomes of lung cancer. 

### 3.4. Anti-Diabetic Drugs

Biguanides and thiazolidinediones are the drug of choice for treating diabetes mellitus. To start with, metformin, a biguanide, has been used as an antidiabetic drug by a direct and indirect mechanism [101]. Metformin acts directly by inhibiting mitochondrial ETC/OxPhos and the consequent activation of AMPK and it has an indirect role by inhibiting hepatic gluconeogenesis, hence lowering systemic insulin levels [102]. Metformin also has an antitumor effect. Knowing that insulin stimulates cellular proliferation and signaling pathways, decreasing insulin levels will indirectly suppress the tumor-stimulating pathways [103]. Additionally, metformin induces cancer cell apoptosis by halting mTOR activity and stimulating the AMPK/LKB1/TORC1 signaling pathway. Further mTOR inhibition, cell cycle arrest, and repression of colony formation ability are achieved by metformin via increasing tumor radiosensitivity through the downregulation of the EGFR/PI3K/Akt signaling pathway [50,104]. The experiments performed to study the effect of metformin on treating lung cancer concluded that metformin was tolerated but did not provide any oncological benefit [51]. In a lung cancer trial where 167 non-diabetic patients with unresectable stage 3 NSCLC maintained on carboplatin and paclitaxel-based chemoradiation either alone or with metformin, the metformin arm failed to provide any significant differences in the rates of survival or distant metastasis [84]. Another drug used to treat diabetes is thiazolidinediones, which functions by activating PPAR-γ (a nuclear receptor), which increases insulin sensitivity and levels of adiponectin resulting in the regulation of glucose metabolism and fatty acid storage [105]. In lung cancer, studies have shown that TZDs prevent the growth of NSCLC cells in vitro [106]. In a xenograft model, TZD blocked tumor progression and, in samples from human lung tumors, decreased PPAR-γ expression worsened the prognosis [105]. Pioglitazone is a PPARγ agonist used commonly for the treatment of type II diabetes. Seabloom et al. recently thought of repurposing pioglitazone for lung cancer treatment via the inhalation route. The authors administered mutated mice with NSCLC cells with aerosolized pioglitazone and the reduction in the size of the adenoma was remarkable [52]. However, insignificant results in terms of inhibiting tumor load and multiplicity were obtained by Zhang et al. when they previously studied the effect of oral pioglitazone in combination with aerosolized budesonide on a lung carcinogenesis mouse model [37]. To conclude, the evidence is inconclusive concerning thiazolidinediones’ role in treating NSCLC and more studies are imperative to determine the downstream effectors of PPAR-γ that mediate the antitumorigenic effects in NSCLC. 

### 3.5. Anti-Microbial Drugs

Bedaquiline, initially FDA-approved as an anti-tuberculosis medication, has shown promising anti-cancer effects. Patil et al. studied the delivery of bedaquiline via inhalation, which demonstrated enhanced anti-NSCLC activity, circumventing the issue of the drug’s poor lung solubility in the aqueous form [107]. These results were also demonstrated by Parvathaneni et al., who showed repurposing bedaquiline as an inhalable cyclodextrin complex is a promising NSCLC treatment [108]. Another antimicrobial medication, Tigecycline, was shown to target NSCLC cells via inhibiting proliferation, inducing the apoptosis of cell lines derived from NSCLC subtypes, and dose-dependently inhibiting the colony formation of highly proliferative and invasive NSCLC subgroups [109]. Tigecycline, through the inhibition of mitochondrial function, may be repurposed as a new targeted NSCLC therapy [109].

Similarly, minocycline may be a repurposed target to be included in NSCLC treatment protocols because it reduces side effects associated with chemoradiation. The local application of minocycline was found to prevent and repair afatinib-induced skin disorders in NSCLC patients [53]. The mechanism was unraveled by histological staining, which showed that minocycline maintained similar EGFR status on the skin of mice treated with anti-EGFR (afatinib) compared to mice not treated with afatinib [53].

Itraconazole is an antifungal shown to inhibit the proliferation, migration, and tube formation of endothelial cells [110]. In a phase I trial in patients with advanced lung cancer, itraconazole was well tolerated in combination with pemetrexed. The overall median progression-free survival was longer in the patients taking itraconazole than in the controls. Even though the mechanism of action of itraconazole as an antineoplastic is not clear yet, it is suggested to be the inhibition of dirtier and the activity against multiple angiogenic stimuli [110]. Furthermore, oral itraconazole’s efficacy in suppressing tumor growth was similar to that of cisplatin in multiple xenograft models of NSCLC [54].

### 3.6. Anti-Helminthic Drugs

Mebendazole, an anti-helminthic that inhibits microtubule synthesis by blocking tubulin polymerization, was shown to have a cytotoxic effect on NSCLC cell lines A549, H1299, and H460 [55]. In vivo, mice treated with mebendazole showed no side effects and had an 80% lower mean colony count as compared to controls [55]. The molecular mechanism involves the stabilization of post-translational p53 and the downstream expression of p21 and MDM2 [55]. Mebendazole can also induce mitotic arrest and apoptosis by depolymerizing tubulin in non-small cell lung cancer cells [111]. Another popular anti-helminthic is niclosamide, which can inhibit glucose uptake and the anaerobic metabolism of cells [112]. Niclosamide has been shown to have an anti-proliferative effect on human lung cancer cells in vivo [113]. Similarly, Piperazine hybrids were also found to have anti-prolific properties against human lung cancer cells [113]. Ivermectin is another anti-helminthic that could be effective in the treatment of NSCLC. Ivermectin’s antineoplastic properties are from its ability to block the canonical WNT (wingless-related integration site) signaling pathway that influences a transcriptional factor of the T-cell factor (TCF) family [114]. Levamisole was shown to arrest the cell in the G0/G1 phase and thus reduce the proliferation of lung cancer cells. Through the restriction of the phosphorylation of c-Jun N-terminal kinase (JNK), levamisole increased the tumor necrosis factor-related apoptosis-inducing ligand (TRAIL)-induced death receptor 4 (DR4)-independent apoptosis rate [56]. 

### 3.7. Anti-Retroviral Drugs

Lopinavir, a protease inhibitor, is an FDA-approved antiretroviral drug for treating HIV, in combination with Ritonavir [57]. This combination is being tested in the treatment of lung cancer. It was shown that Lopinavir and Ritonavir exposure causes cell cycle arrest, decreased cell viability, and apoptosis in lung cancer cells [115]. Efavirenz is another antiretroviral drug that downregulates the synthesis of viral DNA by binding the viral reverse transcriptase [116]. Efavirenz is a candidate for repurposing because it was shown not only to inhibit the progression of lung cancer but also to have a synergic effect in combination with radiation therapy [115,117]. Nelfinavir is another HIV protease inhibitor. It has the potential to be repurposed for NSCLC treatment since it inhibits the proteasome activity in lung cancer cells [118]. Nelfinavir also has a synergic effect with other chemotherapy agents [118]. 

### 3.8. Anti-Malarial Drugs

Atovaquone, an antimalarial drug known to inhibit mitochondrial oxygen consumption, has been shown to reduce tumor hypoxia in patients with NSCLC [58]. Repurposing atovaquone may provide a new radiosensitizer that improves radiation outcomes in NSCLC patients, seeing that tumor hypoxia is a common obstacle in radiation, chemotherapy, and immunotherapy [58]. Pretreatment with chloroquine can oppose nonsmall adenocarcinoma resistance via autophagy inhibition mediated through ROS modulation of the β-catenin pathway [119]. Dihydroartemisinin can lead to the inhibition of NSCLC metastasis via glucose metabolism modulation, specifically by inhibiting the NF-κB pathway, causing dihydroartemisinin to be a promising NSCLC treatment [120]. Quinacrine is an antimalarial that inhibits the FACT (facilitates chromatin transcription) complex, which may be involved in TKI (tyrosine kinase inhibitor) resistance. A phase I clinical trial showed that a combination of erlotinib and quinacrine was well tolerated; however, its efficacy was minimal in advanced NSCLC [121]. 

### 3.9. Other Drugs

Sertraline, an FDA-approved antidepressant, was found to sensitize NSCLC to erlotinib by inducing autophagic flux confirmed by the accumulation of LC3-II and autolysosome formation [59]. The effect of the dual therapy, sertraline, and erlotinib, stems from the regulation of the AMPK/mTOR pathway in NSCLC cells [59]. 

Nitroglycerin is a nitric oxide (NO) donner used to treat angina; however, nitroglycerin can also induce apoptosis, downregulate HIF1alpha, and inhibit angiogenesis [122]. A phase II clinical trial showed that nitroglycerin could improve the response rate to vinorelbine with tolerable toxicity, warranting a phase III trial [123]. The same trial demonstrated that combining vinorelbine and cisplatin with nitroglycerin improved the survival of patients with untreated stage IIIB/IV non-squamous cell lung cancer [123]. 

## 4. Clinical Trials

While using the multiple repurposing approaches mentioned earlier, scientists were able to identify potential targets for repurposing based on the mechanism of action. However, only a few drugs reached the clinical trial stage. Glucocorticoid is the most represented class of medications in the undergoing clinical trials investigating candidate drugs for repurposing in the treatment of NSCLC (Table 2). A phase II trial aims at investigating the combination of prednisone with Afatinib in advanced NSCLC. The study’s outcomes include progression-free survival, overall survival, and response rate (ClinicalTrials.gov; NCT04497584). 

A phase 1/2 study explores the effect of Dexamethasone, a popular glucocorticoid, in reducing the FLT-PET signal in NSCLC (ClinicalTrials.gov; NCT04037462). Similarly, another phase 2/3 trial explores the synergic effect of multiple drugs, including dexamethasone, on the overall survival of advanced stages of NSCLC (ClinicalTrials.gov; NCT05096663).

Metformin, used in the treatment of type 2 diabetes, is explored in a phase 3 trial. The trial assesses the progression-free survival, overall survival, and overall response rate in patients with EGFR mutation NSCLC (ClinicalTrials.gov; NCT05445791).

A phase 1 trial aims to assess the safety of the combination of Statins with PD-1/PD-L1 inhibitors (ClinicalTrials.gov; NC T05636592). Furthermore, the Th1 immune response of the Vancomycin and Stereotactic Body Radiation Therapy combination is explored in an early phase I trial (ClinicalTrials.gov; NCT03546829). Antimalarial drugs are also being investigated in clinical trials. Hence, a phase I trial in locally advanced NSCLC is exploring the safety of Atovaquone by measuring the dose-limiting toxicity (DLT) rate and maximum tolerated dose (MTD) (ClinicalTrials.gov; NCT04648033). Finally, Hydroxychloroquine added to Binimetinib in advanced KRAS mutant NSCLC is being considered in a phase II trial by measuring the objective response rate (ClinicalTrials.gov; NCT04735068).

## 5. Conclusions and Future Directions

Lung cancer remains the main culprit behind cancer-related deaths worldwide, which is why expanding treatment options is paramount. Despite high mortality rates, survival rates are looking more promising. Drugs initially designed for other diseases share similar channels with many lung cancer subtypes. As such, drug repurposing may serve as a fast and cost-effective method of providing new treatment options for lung cancer patients, further boosting survival rates. Drug repurposing bypasses Phase I and II of clinical trials, rendering the drug approval process much swifter than usual. With the ever-growing advances in precision medicine and artificial intelligence, a wider array of drugs can be repurposed in a more directed and strategic manner. This is in hopes of ultimately accounting for patient-specific tumor characteristics and improving survival rates, qualities of life, and overall costs via true personalized medicine. Further studies should be conducted, with in vivo experiments and clinical trials whenever possible, to evaluate potential avenues of drug repurposing within lung cancer treatment.

## Figures and Tables

**Table 1:** Summary table of the drugs that have been repurposed to be used in NSCLC in pre-clinical models.

Refs.	Drug	Original Indication	Cell Lines Targeted	*In Vivo* Studies	Mode(s) of Action	Effect(s)
[34,35,36]	Propranolol	Anti-hypertensive	-	-	Blockade of beta-2 receptors Decreased IL-6 expression	Increased progression-free survival Promoted apoptosis
[37,38]	Telmisartan	Anti-hypertensive	-	-	Blockade of angiotensin II binding to the AT1 receptor Activation of PPARγ	Halted tumor metastasis Decreased the production of VEGF
[39,40,41]	Celecoxib	Nonsteroidal anti-inflammatory	-	SGC-7901 cancer cells	Inhibits cyclooxygenase 2 (COX-2) Inhibits the PDK1/Akt pathway	Decreased cPLA2 and 5-LOX expression Apoptosis demonstrated by the increase in PPAR-γ and p53 expression
[42,43]	Indomethacin	Nonsteroidal anti-inflammatory	-	-	Inhibits cyclooxygenase 1 and 2 (COX-1 and -2)	Apoptosis Initiating the caspase-3 enzymatic activity Upregulate Bax, Bak, and PPAR-ɣ pathway
[44,45]	Atorvastatin	Anti-hyperlipidemic	-	NSCLC A-549 cells	Inhibit β-hydroxy β-methylglutaryl-CoA (HMG-CoA) reductase Inhibit the Akt/mTOR pathway Activates the MAPK pathway	Stimulated autophagy and ferroptosis
[46]	Lovastatin	Anti-hyperlipidemic	A549 lung adenocarcinoma cells	-	Reduces *EGF*-induced phosphorylation of *EGFR* and *Akt* and IR-induced *Akt* phosphorylation Enhances *AMPK* expression and reduces *p53* and the cyclin-dependent kinase inhibitors *p21cip1* and *p27kip1* expression	Redistributed cells from G1-S phase and G2 m phase of cell cycle into apoptosis
[47]	Pitavastatin	Anti-hyperlipidemic	*EGFR* TKI-resistant NSCLC cell lines A549 and Calu6	-	Inhibition of *EGFR/K-RAS* and then of *Akt*	Increased apoptosis
[48,49]	Simvastatin	Anti-hyperlipidemic	Bm7 (R248W) *p53* mutant cells	Balb/C nude mice A459 cancer cells	Increases the caspase-dependent apoptotic pathway Promotes mutant p53 protein degradation Decreases motile activity in lung cancer cells with *p53* missense mutations	Increased apoptosis Inhibition of cell growth, motility, and lipid rafts
[50,51]	Metformin	Anti-diabetic	-	-	Activation of AMPK Inhibit hepatic gluconeogenesis Halting mTOR activity	Increased tumor radiosensitivity through the downregulation of the EGFR/PI3K/Akt signaling pathway
[52]	Pioglitazone	Anti-diabetic	-	benzo[a]pyrene (B[a]P) mouse model	PPARγ agonist	Reduction in the size
[53]	Minocycline	Anti-microbial	-	male ddy mouse	Attaches to the bacterial 30S ribosomal subunit and preventing protein synthesis	Reduced side effects associated with chemoradiation
[54]	Itraconazole	Anti-fungal	-	primary NSCLC xenograft models LX-14 and LX-7	Inhibiting the proliferation, migration, and tube formation of endothelial cells	Longer median progression-free survival Suppression of tumor growth
[55]	Mebendazole	Anti-helminthic	A549, H1299, and H460	WI38	Inhibiting microtubule synthesis	Decreased mean colony count Induced mitotic arrest and apoptosis
[56]	Levamisole	Anti-helminthic	-	-	Agonistic activity at nicotinic receptors in the muscle of parasite	Increased apoptosis rate Reduce the proliferation of NSCLC cells by arresting the cells in G0/G1 phase
[57]	Lopinavir	Anti-retroviral	-	-	Inhibiting HIV-1 protease activity	Causes cell cycle arrest, decreased cell viability, and apoptosis in combination with Ritonavir
[58]	Atovaquone	Anti-malarial	-	-	Inhibiting the mitochondrial electron transport chain at the bc1 complex	Improves radiation outcomes
[59]	Sertraline	Anti-depressant	-	-	Serotonin uptake inhibitor	Induced autophagic flux when combined with erlotinib

**Table 2:** Current clinical trials investigating repurposed drugs for the treatment of NSCLC.

Clinical Trial Number	Phase	Status	Estimated Completion Date	Intervention/Treatment	Patient Population	Patients Enrolled	Primary Outcome Measures	Secondary Outcome Measures
NCT04735068	2	Recruiting	April 2023	Binimetinib + Hydroxychloroquine	Advanced *KRAS* Mutant NSCLC	29	Objective Response Rate 2 years	Progression-free survival (PFS) Number of changes in ctDNA KRAS allelic frequency (blood) Overall survival (OS)
NCT04648033	1	Recruiting	March 2023	Atovaquone	Locally Advanced NSCLC	20	48% dose limiting toxicity (DLT) rate maximum tolerated dose (MTD)	Number of adverse events Correlation between tumor hypoxic volume and tumor hypoxia gene expression Response to treatment
NCT03546829	1	Recruiting	December 2027	Vancomycin + Stereotactic Body Radiation Therapy	Early-Stage NSCLC	40	Th1 immune response	N/A
NCT04980716	3	Recruiting	July 2026	Multiple cardiovascular drugs related to “Golden Triangle”: perindopril, metoprolol Spironolactone trimetazidine hydrochloride statins, antiplatelet aggregation, and nitrate drugs	NSCLC with cardiovascular complications	524	Overall survival	Cumulative incidence of cardiotoxicity events
NCT05636592	1	Recruiting	December 2027	Statins + PD-1/PD-L1 inhibitors	NSCLC	250	Objective response rate, progression-free survival	Overall survival
NCT05445791	3	Recruiting	July 2025	Metformin Hydrochloride	NSCLC with *EGFR* mutations	312	Progression-free survival	Overall Survival, overall response rate
NCT02186847	2	Active not recruiting	April 2024	Metformin + Paclitaxel + Carboplatin + Radiation Therapy	Stage III NSCLC	170	Progression-free Survival	Overall survival, percentage of participants with local–regional progression, distant metastases, and treatment-related grade 3 or higher adverse events
NCT04497584	2	Recruiting	September 2024	Afatinib + Prednisone	Advanced Squamous NSCLC	37	Progression-free survival	Response rate Overall survival
NCT02819024	N/A	Recruiting	November 2023	Dexamethasone	Recurrent, stage III, and stage IV NSCLC	10	Change in tumor SUV max	Change in senescence markers Change in serum dexamethasone concentration
NCT04037462	1/2	Recruiting	January 2025	Dexamethasone	NSCLC	39	Reduction in FLT–PET signal	Response rate
NCT05096663	2/3	Recruiting	December 2027	Cobalamin + Dexamethasone + Docetaxel + Folic Acid + Gemcitabine + Pemetrexed + Ramucirumab	Advanced-, recurrent-, stage III-, stage IV NSCLC	478	Overall survival	Investigator-assessed progression-free survival Duration of response
